# *Yinang* formulation versus placebo granules as a treatment for chronic kidney disease stages III–IV in patients with autosomal dominant polycystic kidney disease: study protocol for a double-blind placebo-controlled randomized clinical trial

**DOI:** 10.1186/s13063-019-3563-5

**Published:** 2019-08-07

**Authors:** Jing Gan, Yansheng Wu, Xuezhong Gong, Yiyi Ma, Shengqiang Yu, Jiandong Gao

**Affiliations:** 1 Department of Nephrology, Shuguang Hospital Affiliated to Shanghai University of Traditional Chinese Medicine, 528 Zhangheng Road, Shanghai, 201203 China; 2 TCM Institute of Kidney Disease, Shanghai University of Traditional Chinese Medicine, 528 Zhangheng Road, Shanghai, 201203 China; 3 Key Laboratory of Liver and Kidney Diseases (Shanghai University of Traditional Chinese Medicine), Ministry of Education, 528 Zhangheng Road, Shanghai, 201203 China; 4 Shanghai Key Laboratory of Traditional Chinese Clinical Medicine (14DZ2273200), No.528 Road ZhangHeng, Shanghai, 201203 China; 5 Department of Nephrology, Shanghai Changzheng Hospital Affiliated to Second Military Medical University, 415 Fengyang Road, Shanghai, 200433 China; 6 Department of Nephrology, Shanghai Municipal Hospital Affiliated to Shanghai University of TCM, 274 Zhijiang Middle Road, Shanghai, 200071 China

**Keywords:** Yinang formulation, Efficacy, Safety, Autosomal dominant polycystic kidney disease, Stages III–IV chronic kidney disease, Randomized controlled trial

## Abstract

**Background:**

Autosomal dominant polycystic kidney disease (ADPKD) is one of the most common potentially life-threatening inherited kidney diseases. It is the fourth most common cause of end-stage renal disease requiring renal replacement therapy. There are few management options for controlling disease progression. Hence, identification of alternative treatments for patients is important. The Chinese herbal *yinang* formulation (YNF), which is derived from a Chinese patent medicine, appears to have a satisfactory effect in treating ADPKD. Because a considerable proportion of ADPKD patients presenting with chronic kidney disease (CKD) stages III–IV are diagnosed with the spleen, kidney deficiency, and blood stasis syndrome according to the diagnostic criteria of traditional Chinese medicine (TCM), we hypothesize that YNF may be a complementary drug for ADPKD patients with the corresponding syndrome. Therefore, we have designed a strict clinical trial to evaluate the safety and efficacy of YNF for ADPKD patients with CKD stages III–IV exhibiting the TCM syndrome of spleen, kidney deficiency, and blood stasis.

**Methods/design:**

This is a multi-center prospective double-blind randomized controlled trial. The total target sample size is planned to be 72 participants, with a balanced treatment allocation (1:1). The experimental intervention will be YNF plus conventional therapy and the control intervention will be a placebo plus conventional therapy for 24 weeks. An additional 24 weeks of follow-up will be conducted after treatment completion. The primary outcome will be the estimated glomerular filtration rate (eGFR). Changes in total kidney volume (TKV), serum creatinine (Scr), blood urea nitrogen (BUN), TCM symptoms, and pain will be the secondary outcomes. Adverse events (AEs) will be monitored throughout the trial.

**Discussion:**

This study will be the first placebo-controlled randomized controlled trial to assess whether YNF plus conventional therapy has a beneficial effect on eGFR, TKV, Scr, and BUN, and whether it can alleviate TCM clinical symptoms, reduce ADPKD-related pain, and reduce the frequency of AEs for ADPKD patients with CKD stages III–IV with the spleen, kidney deficiency, and blood stasis syndrome. The results of this trial may provide an evidence-based recommendation for clinicians.

**Trial registration:**

Chinese Clinical Trials Register, ChiCTR-INR-16009914. Registered on 18 November 2016.

**Electronic supplementary material:**

The online version of this article (10.1186/s13063-019-3563-5) contains supplementary material, which is available to authorized users.

## Background

Autosomal dominant polycystic kidney disease (ADPKD) is one of the most common potentially life-threatening inherited kidney diseases. The incidence of ADPKD worldwide is about 1 in 1000 to 1 in 400 [1]. It is the fourth most common cause of end-stage renal disease (ESRD) requiring renal replacement therapy. It is characterized by accelerated cyst growth resulting in increased total kidney volume (TKV) and renal dysfunction [2, 3], and it affects more than 1.5 million people in China [4] and up to 12 million individuals worldwide [5]. ADPKD is a heterogeneous disorder with two genes identified: PKD1 and PKD2 [6]. The prevalence of autosomal *recessive* polycystic kidney disease is less common than ADPKD, but together with nephrophthaisis is the leading cause of ESRD in childhood [7].

Currently, there are few management options for controlling these diseases. Patients with ADPKD and renal failure are most commonly treated with hemodialysis. The treatment includes non-specific measures that are applicable to all ESRD patients, such as strict blood pressure control, dietary protein restriction, a low salt diet, and statins, which may prevent progression of the disease and reduce cardiovascular mortality [8]. Useful drugs in the management of ADPKD include small-molecule cystic fibrosis transmembrane conductance regulator (CFTR) inhibitors, mammalian target of rapamycin (mTOR) inhibitors, vasopressin V2-receptor (V2R) antagonists, and somatostatin analogues [9]. However, there are a number of limitations with those medications. V2R antagonists and newer agents inhibit pathological pathways in cyst formation. Several preclinical and clinical studies on the V2R antagonist tolvaptan reported evidence of its usefulness in ameliorating the decline of renal function over 1 year in later-stage ADPKD [10]. Since some questions and problems remain (e.g., hepatotoxicity, polyuria, polydipsia, no data on quality of life, and cost-effectiveness) [11], the use of tolvaptan requires careful consideration and balancing of benefits and risks [12]. The lack of specific treatment options for ADPKD makes it difficult for physicians and researchers working in nephrology.

Chinese herbal medicine has been used in the treatment of diseases for thousands of years in China. It is well known that traditional Chinese medicine (TCM) cures ailments based on syndrome differentiation, which is the main characteristic and therapeutic rule of TCM. Clinical information is gathered using the four main diagnostic TCM procedures (observation, listening, interrogation, and pulse-taking) and diagnosis follows TCM criteria. With our many years of clinical practice in the management of ADPKD, we have found that the main pathogenesis of ADPKD is spleen and kidney deficiency (soreness and weakness of the waist and knees, fatigue, cold or numbness of limbs, mental listlessness, lower limb edema, frequent night urination, and sunken pulse) combined with blood stasis (abdominal mass, pain or tingling in the back, subcutaneous petechia, squamous and dry skin, chest tightness, dark purple or dull red tongue with white fur, and strong astringent pulse). The clinical terms for these symptoms are those used in TCM for diagnosis and treatment. The national standard in China for this syndrome was published in February 1997 [13] and was verified by our previous work [14].

The *yinang* formulation (YNF) is derived from a Chinese patent drug originally designated for the clinical treatment of ADPKD patients diagnosed with the spleen, kidney deficiency, and blood stasis syndrome [14]. However, it is not clear whether YNF is effective for ADPKD patients with chronic kidney disease (CKD) stages III–IV. We hypothesized that for patients with ADPKD, YNF could improve the estimated glomerular filtration rate (eGFR), improve serum creatinine (Scr) levels, reduce TKV, reduce the blood pressure, improve symptoms and signs of CKD, reduce ADPKD-related pain, and reduce the number of adverse events (AEs). Thus, a multi-center prospective double-blind placebo-controlled randomized clinical trial was designed to evaluate the safety and efficacy of YNF for CKD stages III–IV in ADPKD patients.

### Aim of the trial

This clinical trial aims to disprove the null hypothesis that YNF granules in an oral dose of 36 g taken twice daily compared to a placebo does not improve the renal function of ADPKD patients at CKD stages III–IV with the spleen, kidney deficiency, and blood stasis syndrome.

## Methods/design

### Trial design

**Fig. 1:**
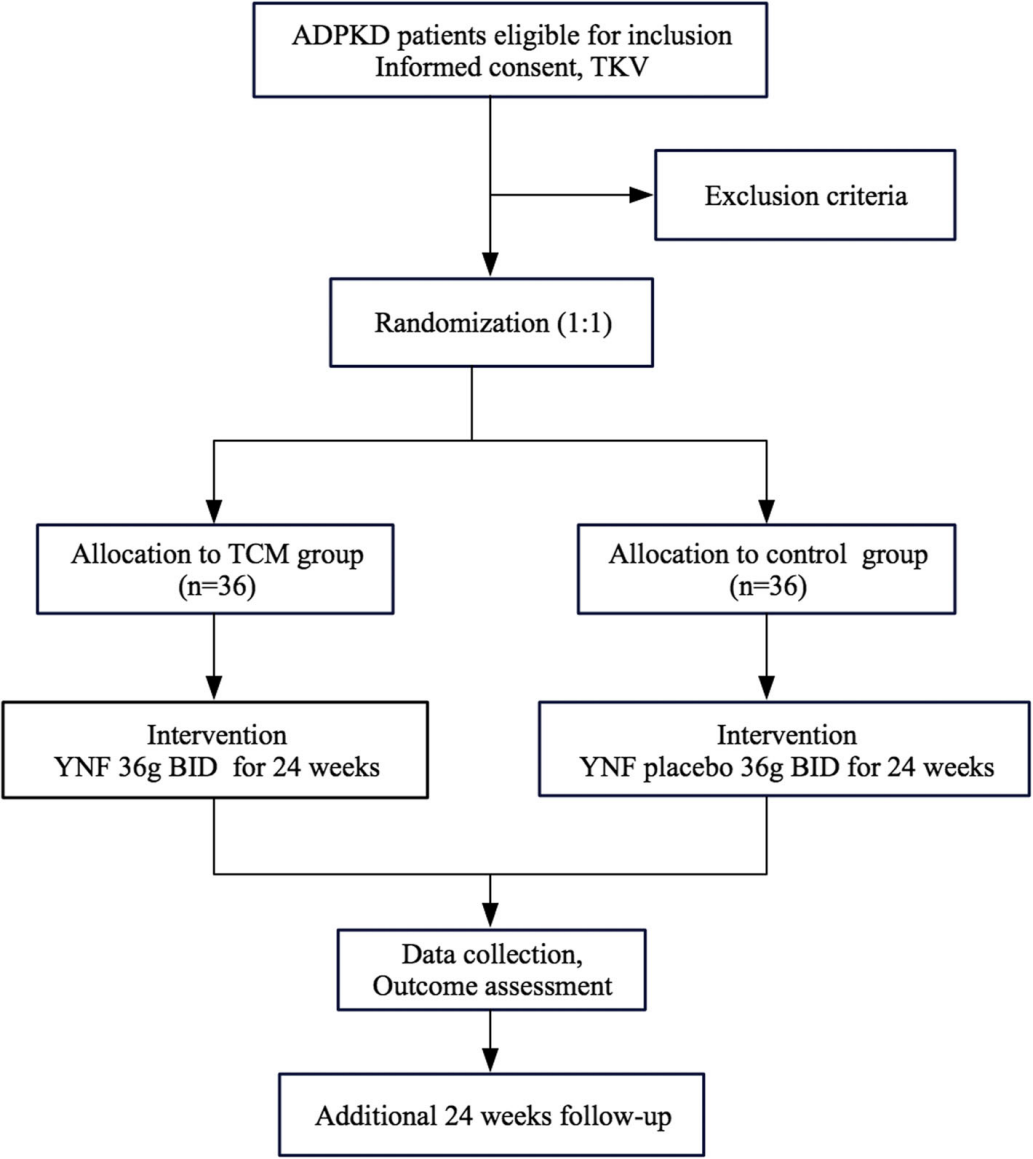
Patient flow in the YNF study for the intention-to-treat analysis. ADPKD autosomal dominant polycystic kidney disease, BID twice daily, TCM traditional Chinese medicine, TKV total kidney volume, YNF *yinang* formulation

This is a multi-center randomized double-blind placebo-controlled clinical trial lasting 24 weeks. An additional 24 weeks of follow-up will be conducted after treatment completion. The design of the trial follows a strict scientific clinical research methodology and complies with principles of the Declaration of Helsinki and the guidelines for good clinical practice (Fig. 1).

### Sample size calculation

The sample size was based on the primary outcome of a change in baseline eGFR. To determine the sample size, we reviewed unpublished data for eGFR changes for 22 ADPKD outpatients at CKD stages III–IV. The mean value of eGFR was 26.71 mL/min/1.73 m^2^, and the standard deviation was 8.53 mL/min/1.73 m^2^. It was assumed that eGFR for the YNF group would be significantly higher than for the control group by more than 6 mL/min/1.73 m^2^ after 24 weeks. If α = 0.05 and the test efficacy 1 – *β* = 0.80, then the sample size

$$ {n}_1=\kern0.5em \kappa {n}_2 $$

where

$$ {n}_2=\frac{\left({\mathrm{Z}}_{\alpha /2}+{\mathrm{Z}}_{\beta}\right){}^2\kern0.5em \sigma {}^2\kern0.5em \left(1\kern0.5em +\kern0.5em 1/\kappa \right)}{\varepsilon^2}. $$

Thus, *n*_1_ = 64, with 32 in each treatment group. Considering a 10% dropout rate, the planned sample size for randomization was increased to 72. Participants will be randomly assigned in a ratio of 1:1. Each of the three participating hospitals will recruit 24 patients, 12 in the experimental group and 12 in the control group.

### Study setting and recruitment

This study will recruit 24 outpatients at each of three trial sites in Shanghai in mainland China: (1) Shuguang Hospital affiliated to Shanghai University of TCM, (2) Shanghai Changzheng Hospital affiliated to the Second Military Medical University, and (3) Shanghai Municipal Hospital affiliated to Shanghai University of TCM. Advertisements to encourage people to enroll in the clinical trial will be posted on the study's web page.

### Participants

The study plans to enroll 72 patients diagnosed with ADPKD at CKD stages III–IV and with the spleen, kidney deficiency, and blood stasis syndrome. To be enrolled in the trial, potential participants must satisfy the inclusion and exclusion criteria listed below.

### Diagnostic criteria for ADPKD

Participants are diagnosed with ADPKD based on a known family history, imaging features, or genetic testing. No distinction will be made between those with mutations of PKD1 or PKD2. For patients with ADPKD, eGFR is within the range 15 to 60 ml/min/1.73 m^2^. Patients have abnormal laboratory tests, and loss of renal structure and endocrine function for more than 3 months. It is an irreversible and chronic progressive disease [15].

### Diagnostic criteria for TCM syndrome differentiation

According to the *Guidelines for Clinical Research of Chinese Medicine (New Drug)* [16], a patient has to present with at least two of the primary symptoms and more than two of the secondary symptoms listed below to be diagnosed with the spleen, kidney deficiency and blood stasis syndrome.

Primary signs and symptoms:

- abdominal mass
- soreness and weakness of the waist and knees
- pain or tingling in the back
- fatigue
- cold or numbness of limbs

Secondary symptoms:

- mental listlessness
- lower limb edema
- subcutaneous petechia
- squamous and dry skin
- chest tightness
- frequent night urination
- dark purple or dull red tongue with white fur
- weak, sunken, or strong astringent pulse

### Inclusion criteria

- adult subjects with a diagnosis of ADPKD as stated above and with a TCM diagnosis of the spleen, kidney deficiency, and blood stasis syndrome
- aged 18–75 years
- 15 mL/min/1.73 m^2^ ≤ eGFR < 60 mL/min/1.73 m^2^
- not receiving renal replacement therapy
- not suffering from chronic hepatitis, hepatic dysfunction, cardiovascular diseases, or any other life-threatening conditions
- negative urine or serum pregnancy test within 24 h prior to administration of YNF and agrees to use contraception throughout the study and for 6 months after

### Exclusion criteria

- ADPKD is accompanied by proteinuria (> 1 g/d)
- diabetic patients
- unable to or do not consent to participate in the study
- suffering from chronic hepatitis, drug-induced hepatic dysfunction, or another type of hepatic dysfunction
- recent participation in another clinical trial
- probable, highly likely, or definite allergy to any of the ingredients in the test drug
- contraindications to magnetic resonance imaging (MRI)
- taking medications likely to affect ADPKD outcomes

### Participant withdrawal criteria

Participants will be withdrawn from the study if

- They violate any of the key inclusion or exclusion criteria.
- They refuse to continue to participate or withdraw their consent.
- They undergo a serious adverse event (SAE).
- They have unbearable ADPKD-related pain and choose cyst decompression as a surgical intervention during the study.
- The principal investigator or co-investigators judge that they need to be withdrawn from the study.
- A hepatocellular injury is detected, indicated by alanine transaminase (ALT) or aspartate transaminase (AST) levels > 3 × ULN and total bilirubin > 2 × ULN, where ULN is the upper limit of normal.
- They have hematuria during treatment.

### Randomization

The central randomization list was generated by an independent statistician using SPSS (version 24.0, IBM, NY, USA). Altogether, 36 patients will be assigned to the intervention group and to the control group using a balanced block randomization with 6 blocks. We will randomly select blocks of size 4. Telephone-based randomization will be performed at each coordinating center by an independent physician who is not engaged in recruitment, treatment, or assessment. Participants will be randomly assigned in each site to the YNF group and the placebo group in a ratio of 1:1. After screening, if the patient agrees to participate and voluntarily signs the informed consent form (Additional file 2), the independent physician in each trial site will sequentially assign a random number to them.

### Blinding

This clinical trial will be double blind. The physicians, investigators, co-investigators, clinical trial pharmacists, and patients are all blinded to the allocation, except for the independent statistician who generated the randomization.

The placebo granules will be indistinguishable from the YNF granules in shape, size, color, and packaging. Thus, all clinicians and participants will be masked. The packaging will be labeled with sequential random numbers in advance by a pharmaceutical company according to the randomization list sent by the independent statistician. The independent statistician will go to the pharmaceutical company to check the packaging when the drugs are being labeled. At each trial site, based on their allocation, a patient will be given the package of YNF or the placebo with the lowest number by a designated clinical trial pharmacist who is not involved with the study and is blind to the allocation.

Unblinding will be done according to the standard operating procedure of the contract research organization [17]. For each subject, an emergency plan and their treatment plan will be sealed in an opaque envelope, to allow unblinding if there is a serious adverse reaction and possibly to ensure the correct treatment method was administered. If an emergency occurs, the principal investigator will ask an independent physician to unblind the patient and the incident will be reported to the independent safety monitoring board.

### Intervention

According to the guideline for the management of ADPKD [18], drugs used in the treatment of hypertension, hematuria, concurrent infections, and other diseases will be used reasonably for patients in both groups as basic therapy, except for Chinese patent drugs. The name and dosage of any drug administered will be recorded. In addition to their standardized Western medicine, participants randomized to the treatment group will be administered YNF as an oral dose of 36 g twice daily 1 h after breakfast and dinner for 24 weeks. Those in the control group will receive the placebo as an oral dose of 36 g twice daily 1 h after breakfast and dinner for 24 weeks. Both groups will receive behavioral intervention and health education, such as diet instructions. All co-investigators and patients will be educated about drug administration methods.

The YNF granules (10% YNF) and the placebo granules will be manufactured by Jiangsu Tianyin Pharmaceutical Co. Ltd. (1 Xin Sheng Street, Jiangyin High-tech Zone, Jiangsu) according to good manufacturing practice. The composition and action of each herb are summarized in Table 1.

**Table 1:** Composition and action of *yinang* formulation in Chinese herbal medicine

Ingredient	Granule dose	Action (TCM)	Pharmaceutical action
Rhizoma sparganii (*sanleng*)	10 g	1. Regulate qi 2. Relieve pain 3. Promote blood circulation 4. Alleviate stagnation	1. Inhibit platelet aggregation and thrombosis
Ranunculus ternatus Thunb (*maozhuacao*)	30 g	1. Dissipate phlegm 2. Dispel stasis 3. Detoxify 4. Detumescence	1. Anti-inflammation 2. Anti-tumor
Spina gleditsiae (*zaojiaoci*)	6 g	1. Detoxify 2. Disperse swelling	1. Anti-tumor
Rhizoma drynariae (*gusuibu*)	10 g	1. Reinforce the kidneys 2. Strengthen bones 3. Relieve pain	1. Prevent osteoporosis 2. Anti-inflammatory
Astragalus (*huangqi*)	30 g	1. Tonify qi 2. Diuresis	1. Improve the immune function 2. Anti-oxidant 3. Increase resistance to radiation 4. Anti-tumor
*Codonopsis pilosula* (*dangshen*)	30 g	1. Invigorate the spleen 2. Replenish qi	1. Regulate the central nervous system 2. Enhance immune function 3. Regulate gastrointestinal motility 4. Cardiovascular protective effect
Angelica sinensis (*danggui*)	10 g	1. Enrich the blood 2. Invigorate the circulation of blood	1. Anti-atherosclerosis 2. Bacteriostasis 3. Anti-hypoxia 4. Regulation of immune function 5. Inhibit platelet aggregation
Paeoniae radix (*chishao*)	15 g	1. Activate blood circulation to dissipate blood stasis	1. Inhibition of platelet and erythrocyte aggregation 2. Anti-atherosclerosis 3. Anti-tumor 4. Protect the heart and liver 5. Anti-coagulation and anti-thrombosis
Radix paeoniae alba (*baishao*)	10 g	1. Nourish the blood 2. Reduce yin 3. Soothe the liver 4. Relieve pain 5. Stabilize yin and yang in the liver	1. Anti-inflammation 2. Pain relief 3. Liver protection
*Duchesnea indica* (*shemei*)	10 g	1. Clear away heat and toxic material 2. Activate blood circulation to dissipate blood stasis	1. Anti-oxidant
Hedyotis diffusa willd (*baihuasheshecao*)	15 g	1. Dissolve phlegm 2. Detoxification 3. Detumescence	1. Anti-inflammation 2. Anti-tumor 3. Regulation of immune function
Cowherb seed (*wangbuliuxing*)	15 g	1. Activate blood circulation to dredge collaterals 2. Detumescence 3. Relieve pain	1. Inhibit microcapillary proliferation 2. Inhibit migration and adhesion of endothelial cells
Semen brassicae (*baijiezi*)	15 g	1. Activate qi 2. Activate blood circulation 3. Dredge collaterals 4. Relieve pain	1. Anti-hepatic fibrosis 2. Regulation of TGF-β1/Smad, NF-κB, and AKT signaling pathways 3. Reduction of extracellular matrix deposition
Plantaginis seeds (*cheqianzi*)	15 g	1. Clear heat 2. Diuresis 3. Permeate dampness 4. Promote circulation 5. Clear eyesight 6. Expectorant	1. Eliminate oxygenic free radicals 2. Anti-oxidation in lipid metabolism 3. Anti-oxidation
*Polygonum cuspidatum* (*huzhang*)	10 g	1. Relieve coughing 2. Resolve phlegm 3. Dispel wind dampness 4. Remove fluid 5. Dissipate blood stasis 6. Relieve pain	1. Anti-bacterial and anti-viral 2. Protect the cardiovascular system 3. Anti-inflammation and analgesic 4. Liver protection 5. Enhance immunity 6. Anti-tumor
*Centella asiatica* (*jixuecao*)	30 g	1. Clear heat 2. Disperse dampness 3. Detoxify 4. Reduce swelling	1. Improve the microcirculation 2. Relieve pain 3. Anti-oxidant 4. Anti-bacterial 5. Anti-inflammation 6. Anti-fibrous tissue proliferation
Prepared rhubarb (*dahuang*)	10 g	1. Alleviate accumulation 2. Clear heat 3. Detoxify 4. Remove blood stasis 5. Dredge meridians	1. Promote defecation 2. Anti-bacterial 3. Protect the liver and gallbladder 4. Lower blood pressure 5. Lower blood lipid levels

*TCM* traditional Chinese medicine

### Measurement items and time points of data collection

Relevant examinations will be performed to collect patient data at 24 weeks before baseline, at baseline, every 4 weeks from 4 to 24 weeks during treatment, and an additional follow-up evaluation will be performed at visit 9 (48 weeks). General information collected will include name, age, gender, ID number, date of birth, home address, contact information, history of present illness, past medical history, family history, personal life history, menstrual history, marital history, allergy history, social history, physical examination results, and laboratory examination results.

There is evidence that age-related TKV can reflect the progress of ADPKD. An MRI scan can accurately measure renal blood flow and evaluate renal cyst compression, which is associated with pain, hypertension, hematuria, albuminuria, and loss of renal function. TKV combined with age and renal function can be used to evaluate the risk of progress to ESRD [19]. The T2 shortening imaging technique of MRI with no radiation damage, no use of gadolinium as a contrast agent, and no risk of nephrogenic systemic fibrosis is recommended and will be used in this study [20]. The outcome is the percentage semi-annual change in TKV measured by MRI, which will be performed at visit 1, visit 2 (24 weeks after visit 1), and visit 8 (24 weeks after visit 2) to observe the growth in kidney volume before and after the intervention.

Items to be measured and the time points of data collection are listed in Table 2.

**Table 2:** SPIRIT table: Measurement items and time points of data capture

Items	Screening	Baseline	Treatment						Follow-up
	Visit 1	Visit 2	Visit 3	Visit 4	Visit 5	Visit 6	Visit 7	Visit 8	Visit 9
	−24 weeks	0 weeks	4 weeks	8 weeks	12 weeks	16 weeks	20 weeks	24 weeks	48 weeks
General									
Informed consent	×								
Inclusion and exclusion criteria	×								
Collect general information	×								
General physical examination	×								
Urine hCG (only women)	×								
Medical and drug use history	×								
Concomitant disease and treatment	×								
Combined medication	×	×	×	×	×	×	×	×	×
Effectiveness observation									
Vital signs	×	×	×	×	×	×	×	×	×
TCM symptoms	×	×	×	×	×	×	×	×	×
Pain evaluation	×	×	×	×	×	×	×	×	×
Renal function (eGFR, Scr, BUN)	×	×	×	×	×	×	×	×	×
MRI (TKV)	×	×						×	
Blood electrolytes	×	×			×			×	×
Urine β2-MG, mALB/Cr, RBP	×	×						×	×
Safety observation									
Blood test ^#^	×	×			×			×	×
Urine test	×	×	×	×	×	×	×	×	×
Stool routine and occult blood test	×	×			×			×	×
Liver function (ALT, AST, γ-GT)	×	×			×			×	×
Electrocardiogram	×	×						×	×
Other data									
Adverse events			×	×	×	×	×	×	
Allocation		×							
Drug distribution		×	×	×	×	×	×		
Drug recycling			×	×	×	×	×	×	
Drug count			×	×	×	×	×	×	
Research conclusion								×	×

Each visit allows a window of 3 days

# The blood test assesses the red blood cell count, white blood cell count, hemoglobin level, and platelet level

*eGFR* estimated glomerular filtration rate, *hCG* human chorionic gonadotrophin, *Scr* serum creatinine, *BUN* blood urea nitrogen, *β2-MG* β2-microglobulin, *mALB/Cr* microalbumin/creatinine, *MRI* magnetic resonance imaging, *RBP* retinol-binding protein, *ALT* alanine aminotransaminase, *AST* aspartate aminotransaminase, *γ-GT* gamma glutamyl transpeptidase, *TCM* traditional Chinese medicine, *TKV* total kidney volume

### Outcomes

The primary outcome of this trial will be the eGFR using the CKD Epidemiology Collaboration formula [21]. Secondary outcomes include changes in TKV, Scr, blood urea nitrogen (BUN), TCM symptoms, and pain. A composite outcome looks at the incidence rates of all possible safety events between two groups. AEs will be monitored throughout the trial and several biological indicators (blood and urine tests, electrocardiograms, and liver function test) will also be closely watched.

### Measurement scale for TCM symptoms

The measurement scale for TCM symptoms recommended by the *Guidelines for Clinical Research of Chinese Medicine (New Drug)* [16] will be used for ease of assessment. Each of the primary symptoms necessary for the diagnosis of the spleen, kidney deficiency, and blood stasis syndrome will be scored 0, 2, 4, or 6, while secondary symptoms will be scored 0, 1, 2, or 3. The scores will be summed to yield a total score for each set of symptoms for each patient. The total primary symptom score cannot exceed 18 and the total score for secondary symptoms cannot exceed 33 for a patient. The two totals will be added together and any change in this score will be calculated for each patient and used to calculate an efficacy indicator (EI) for the evaluation of treatment efficacy:

$$ \mathrm{EI}=\left(\mathrm{Total}\ \mathrm{symptom}\ \mathrm{score}\ \mathrm{at}\ \mathrm{baseline}-\mathrm{Total}\ \mathrm{symptom}\ \mathrm{score}\ \mathrm{post}-\mathrm{treatment}\right)/\mathrm{Total}\ \mathrm{symptom}\ \mathrm{score}\ \mathrm{at}\ \mathrm{baseline}\times \kern0.37em 100\% $$

The degree of symptom improvement will be presented in four categories ranging from full recovery (EI ≥ 90%), good recovery (90% > EI ≥ 70%), and modest recovery (70% > EI ≥ 30%) to no recovery (EI < 30%).

### Pain evaluation scale

Pain is the most common complaint of patients with ADPKD. Causes of pain are multiple but include cyst enlargement, cyst rupture, and secondary infection [22]. Good remission of ADPKD-related pain (low back pain, abdominal pain, headache, chest pain, and leg pain) can alter a patient’s quality of life. The visual analog scale (VAS) is a 10-cm horizontal line anchored by two extremes. It will be used to measure ADPKD-related pain [23]. Patients will be instructed to complete a pain diary for the day at each visit. According to this scale, 0 cm means no pain or no discomfort, whereas the 10-cm end indicates the worst pain or extreme discomfort. The baseline will be the VAS score at visit 2. The difference in pre- and post-treatment VAS scores will be a secondary outcome. At the final visit, patients will complete a global assessment of their overall responses after the trial. The global assessment [24] is a five-point scale: 0 = worsened, 1 = no change, 2 = slightly improved, 3 = improved, and 4 = significantly improved.

### Statistical analysis

A formal and detailed statistical analysis plan will be formulated before the database is locked. The baseline characteristics of both groups, such as gender, age, and smoking status, will be compared by either a χ^2^ test or Student’s *t*-test. Continuous variables will be presented as means ± standard deviations or medians, and differences in such variables will be analyzed using an independent *t*-test or the Wilcoxon signed-rank test. A repeated measurement of variance analysis model of these outcomes will be built.

The difference in eGFR will be tested using two-way ANOVA with repeated measures. Categorical variables will be expressed as numbers and percentages, and differences in such variables will be calculated and compared using a χ^2^ test and Fisher’s exact test. All statistical tests will be two-sided, and the level of significance will be set to *P* < 0.05. Multiple imputation will be used to handle missing data. All patients enrolled will be included in the primary analysis in accordance with the intention-to-treat principle. The statistical analysis will be performed in a blind manner by an independent statistician using SPSS (version 24.0, IBM, NY, USA). No additional analyses or an interim analysis are scheduled.

### Data and safety monitoring

An independent data and safety monitoring board comprising cardiovascular surgeons, anesthesiologists, and statisticians will oversee all aspects of the study. The board will meet twice during the trial to monitor safety. It will make recommendations on study progress and performance, identify any major adverse outcomes or AEs due to the therapy, and give advice regarding whether the study should continue or if there should be a protocol change.

In this study, AEs are defined as any negative or unintended clinical manifestations that occur after the start of the study, regardless of medication. Any unexpected symptoms and signs, feelings of discomfort, or occurrence of AEs in patients during the trial will be recorded using medical diagnostic terminology on a case report form with detailed symptoms, time of occurrence, duration, severity, possible causal relationships, actions taken, results, and other relevant information. If an AE occurs, the patient will be monitored until their AE disappears. A follow-up investigation will be conducted, and the results will be recorded in case the intervention is halted due to the AE. The chief principal investigator (JDG) is responsible for reporting all AEs to the board and the regulatory authorities within 24 h.

The following are possible safety events relating to the intervention:

- decrease of eGFR by ≥50%
- Scr doubled since the last blood test
- progression to CKD stage V
- serious cardiovascular or cerebrovascular events
- heart rate < 40 bpm
- any unexpected event that the investigator believes could be attributed to the intervention

SAEs include any of the following:

- fatal or life-threatening complications
- death or persistent or significant disability
- hospitalization or significant medical intervention to prevent a serious outcome
- any events that investigators examine are significant hazards or harm to the participants

Stopping rules for the trial:

- If an insufficient number of patients is enrolled.
- If the allocation codes are leaked during the trial or if more than 20% of the envelopes with emergency plans are opened, which would mean that the blinding has been nullified.
- Occurrence of SAEs that are likely to be related to the intervention drugs in the trial, which may indicate there are serious problems with the safety of the drug.

### Data entry and quality control of data

Participant adherence to the protocol will be monitored by interviews at study check-up visits, which will promote retention and completion of follow-up assessments.

After verification of the content of the written case report forms, the data will be input independently into a database by two full-time research staff. To maintain data quality, all investigators will receive centralized training before the trial begins. Personal information (such as name, age, gender, ID number, date of birth, home address, contact information) will be kept in a locked storage unit according to standard guidelines. The quality control of the data will be assessed by the data and safety monitoring board three times at each trial site. The board will check that study procedures have been followed correctly according to the approved protocol. They will compare the data in the database with the source documents to evaluate its accuracy, completeness, and authenticity. The data and safety monitoring board will meet when the first participant enrolls, when 50% of the participants have been recruited, and when the last participant enrolls.

### Ethics approval

This study has been approved by the international review board of each participating hospital. The trial was registered with the Chinese Clinical Trials Register (ChiCTR-INR-16009914) on 11 November 2016 (http://www.chictr.org.cn/showproj.aspx?proj=16783). Only clinicians with relevant qualifications will act as principal investigators. Written informed consent for the collection and use of participant data and biological specimens will be obtained from individual participants or authorized surrogates prior to enrollment. Personal information about potential and enrolled participants will not be disclosed to any third party before, during, or after the trial.

## Discussion

There are few management options for ADPKD. In East Asia, Chinese herbal medicine is one of the most common treatments. In TCM, the spleen and kidneys govern the movement and transformation of qi and fluid and these organs cooperate with each other to participate in the metabolism of water. A functional disorder of the spleen or kidneys would lead to qi stagnation and blood stasis, resulting in abdominal mass and fatigue. Therefore, nourishing the kidneys and spleen and removing blood stasis is an important aspect of treatment.

YNF is an improved version of a herbal prescription based on the therapeutic work of the late TCM practitioner Pingdong Zheng, who enjoyed nationwide fame for treating patients with chronic renal failure [25]. Compared with the original patent prescription [14], YNF is better in two ways. First, pangolin powder, which is expensive, was substituted by spina gleditsiae. Second, TCM granules are used instead of TCM slices to ensure the quality requirements in term of drug concentration and effectiveness of the ingredients.

YNF is a combination of 17 herbal ingredients, which have a synergistic effect of nourishing the kidneys and spleen and alleviating blood stasis according to preclinical study evidence [14]. It is recommended for treating a variety of indications, including abdominal mass, soreness and weakness of the waist and knees, pain or tingling in the back, fatigue, cold or numbness of limbs, and mental listlessness. The potential mechanism of YNF in the treatment of ADPKD needs to be further explored. It has been reported that some of the herbs in YNF have pharmacological effects, such as inhibiting platelet and erythrocyte aggregation and improving the microcirculation [26, 27], relieving pain [28], anti-inflammation [29], anti-fibrous tissue proliferation [30, 31], anti-oxidant [32, 33], and anti-tumor [34] activities. Such activities may contribute (1) to the inhibition of cyst growth and cell proliferation, (2) to reducing the compression of the renal parenchyma, which is good for relieving ADPKD-related pain, and (3) to down-regulating Scr and BUN levels to improve renal function. Understanding which ingredients of YNF alleviate the symptoms of ADPKD requires further research. YNF has been used in clinical practice for several years and no safety concerns have been raised. The common adverse effects of YNF, such as diarrhea and increased urine output, appear to be mild and self-limited. The favorable safety profile of YNF increases its acceptability with the general population.

In view of the current evidence base, we have designed this placebo-controlled randomized controlled trial to test the efficacy and safety of YNF for ADPKD patients with CKD stages III–IV with the spleen, kidney deficiency, and blood stasis syndrome. This protocol complies with the SPIRIT 2013 [35] statement and SPIRIT 2013 explanation and elaboration [36], which cover scientific, ethical, and safety issues (Additional file 1). For patients with ADPKD, the findings of this study may help to improve their clinical symptoms and renal function, and slow cyst growth. We expect that this trial may provide preliminary evidence for the efficacy of YNF in treating ADPKD, which would be useful for researchers, practitioners, and patients.

There are some limitations to this study. First, considering the longer term, it was not feasible to measure the rates of more clinically important outcomes, such as renal replacement therapy or transplant. Second, this study is being performed in Shanghai, China, and it is uncertain whether the relative effects of YNF would be similar in other ethnic groups. Third, which ingredients in YNF contribute to the treatment effect needs further research and exploration.

### Trial status

The research strategy and study protocol were developed between October 2017 and June 2019. From October 2018 to July 2019, patients were screened and TKV was measured by MRI 24 weeks before the baseline. The follow-up visits and data analysis will take place from July 2019 to December 2020.

## Additional files

Additional file 1: SPIRIT 2013 checklist. Recommended items to address in a clinical trial protocol and related documents. (PDF 127 kb)

Additional file 2: Model consent form given to participants and authorized surrogates. (ZIP 39 kb)

## Data Availability

Does not apply.

## Abbreviations

ACEI: Angiotensin-converting enzyme inhibitor
ADPKD: Autosomal dominant polycystic kidney disease
AE: Adverse event
ALB: Serum albumin
ALT: Alanine aminotransaminase
ARB: Angiotensin receptor blocker
AST: Aspartate aminotransaminase
BID: Twice daily
BUN: Blood urea nitrogen
CKD: Chronic kidney disease
eGFR: Estimated glomerular filtration rate
EI: Efficacy indicator
ESRD: End-stage renal disease
hCG: Human chorionic gonadotrophin
mALB/Cr: Microalbumin/creatinine
MRI: Magnetic resonance imaging
PKD: Polycystic kidney disease
RBP: Retinol-binding protein
SAE: Serious adverse event
Scr: Serum creatinine
TCM: Traditional Chinese medicine
TKV: Total kidney volume
ULN: Upper limit of normal
V2R: Vasopressin V2-receptor
VAS: Visual analog scale
YNF: *Yinang* formulation
β2-MG: β2-microglobulin
γ-GT: Gamma glutamyl transpeptidase
