# Supplementary material for: Yinang formulation versus placebo granules as a treatment for chronic kidney disease stages III–IV in patients with autosomal dominant polycystic kidney disease: study protocol for a double-blind placebo-controlled randomized clinical trial
Source: Trials. 2019 Aug 7;20:481. doi: 10.1186/s13063-019-3563-5 (PMC6686499; doi:10.1186/s13063-019-3563-5)
Supplement: Supplementary file 2 — Model consent form given to participants and authorized surrogates. (ZIP 39 kb) [file 13063_2019_3563_MOESM2_ESM.zip › Additional file 2-Consent form in ChineseR2.doc]

知 情 同 意 书

（第 03版， 2018年9月18日）

受试者姓名 ： ________________

联 系 地址 ： ________________

联 系 电 话： ________________

研究中心名称： _______________

**知情同意书·告知页**

**尊敬的___________ 先生/女士：**

我们真诚地邀请您参加“抑囊方治疗多囊肾CKD3-4的临床研究”。本研究由上海中医药大学附属曙光医院肾病科主持进行，上海长征医院、上海市中医医院为本研究分中心之一。在您同意参加本项研究之前，请您仔细阅读这份知情同意书，它将提供给您本研究背景、目的、方法、试验过程中给您带来的益处和可能产生的风险或者不变以及您的权益保护等内容。本知情同意书提供给您的信息可以帮助您决定是否参加此项研究。如有任何疑问可以向该项目的研究者咨询，以确保您充分理解有关的内容。如果您同意参加本研究，请您签署知情同意书，并且保存一份双方签字的知情同意书。本研究方案已通过上海长征医院、上海市中医医院伦理委员会批准。

**1、为什么要参加这项研究？**

常染色体显性遗传多囊肾病(autosomal dominant polycystic kidney disease ，ADPKD) 简称多囊肾（polycystic kidney disease，PKD），是人类最常见的遗传性肾病之一，其在全世界发病率约为1/1000～1/400，已成为进入终末期肾病的第4位病因,目前尚无有效药物控制疾病进展，多数患者通过血液透析维持生存，给患者和社会带来沉重医疗和经济负担。成人多囊肾为常染色体显性遗传病，由基因突变引起，目前已知引起成人多囊肾的突变基因为PKD1和PKD2，与其他人种相比，因基因组多态性的不同，我国汉族人种基因序列存在自身的特点，其多囊肾病基因的突变也同样存在自身的规律，但相关研究还在起步阶段。ADPKD发病年龄一般在30-40岁，60岁患者中，50％以上进入终末期肾衰竭，占终末期肾衰竭病因的10%左右。血透病人中约2.7％由于ADPKD引起，腹透病人中约3.8％由于ADPKD引起。欧美国家和我国上海地区的血液透析患者资料数据库均显示，PKD是导致终末期肾病的第4位病因。美国PKD患者每年有近17000人进入终末期肾衰，医疗费用高达6亿美元。

作为基因突变导致的遗传性疾病，治疗ADPKD的理想方法是采用正常基因替换突变基因，纠正蛋白功能异常，但目前临床上尚无法实现。此外，对于多囊肾靶向治疗制剂如抑制细胞增殖和抑制囊液分泌药物等尚处于研发阶段。21世纪以来肾素血管紧张素抑制剂成为治疗多囊肾的可能有效药物。肾间质纤维化也是影响多囊肾进展的重要危险因素，而西医目前尚无有效药物对抗肾脏纤维化的进展。除了保守治疗以外，肾脏引流联合去顶减压手术也是治疗多囊肾的常用方法，一项对169例PKD患者实施去顶减压术的报道表明，选择性的对一、二期病人进行去顶减压手术对改善症状,降低血压、延缓肾功能衰竭具有良好的效果。对于三期病人则在术前术后无差异。因此，多囊肾依然是医学界需要攻克的难题。

我们采用抑囊方补肾健脾、活血化瘀软坚、利水治疗多囊肾病。抑囊方以补肾健脾利水，活血化瘀软坚为主导思想。全方气血并治，攻补兼施，补而不滞，攻而不伤，故收良效。我们最近的一项对35例多囊肾患者临床观察证实，经一般处理联合抑囊方治疗后患者中医证候改善率达97.1%。证候总积分在治疗3 个月、6 个月后，较0 周纵向比较均有明显改善，组内两两比较亦差异有统计学意义(P<0.01) ;6 个月结束时，治疗前后尿NAG、尿RBP、尿微量白蛋白/尿肌酐均有所下降，其改善程度差异具有统计学意义(P<0.05) 。中药复方抑囊方不仅可以改善多囊肾患者临床症状，而且对改善多囊肾患者肾小球、肾小管功能相关指标也有较好疗效。

本研究的目的是为了探索寻找延缓多囊肾进展的新药和新方法，评价抑囊方治疗多囊肾CKD3-4期的有效性和安全性，其研究结果将用于形成临床新药。

本研究项目是由上海申康医院发展中心/促进市级医院临床技能与临床创新三年行动计划资助（课题编号：16CR3021A ）。本研究项目由上海中医药大学附属曙光医院伦理委员会、上海长征医院伦理委员会、上海市中医医院伦理委员会审议，是遵从中国国家相关法规和赫尔辛基宣言等保护受试者权益的伦理原则的。

**2、哪些人可以参加这项研究？**

（1）纳入标准

①符合多囊肾及中医证候诊断标准；

②年龄18-75岁；

③肾功能15ml/min≤GFR＜60ml/min。

④未行肾脏替代治疗；

⑤无心血管、肝和造血系统等严重原发性疾病；

⑥药物治疗24h以内，尿液或血清妊娠检查阴性，试验期间和试验六个月后同意避孕。

（2）排除标准

①ADPKD伴有蛋白尿(> 1 g / d)和糖尿病患者。

②认知障碍。

③慢性肝炎患者,药物引起的肝功能障碍和其他肝障碍。

④近期参与另一个临床试验。

⑤可能，极有可能或明确的药物成分过敏测试。

⑥患者有磁共振成像禁忌症。

⑦患者不同意参与这项研究。

⑧病人服用的药物可能影响ADPKD的结果。

**3、多少人参加这项研究？**

研究预计招募72名符合条件的受试者参加该项研究，这些患者将按照1:1比例随机分配到基础治疗联合抑囊方组或者基础治疗联合中药模拟剂组，模拟剂在外形包装、颗粒形态色味等方面保持与抑囊方颗粒剂一致，包含抑囊方十分之一的药物含量。您有可能进入抑囊方试验组或中药模拟剂对照组（进入任意一组的可能性是50%），但仅能进入到其中一组进行治疗和评价。而不论您进入到哪一组，都会针对您的病情给予合适的基础及对症治疗。

**4、研究是怎样进行的？**

我们首先会筛选临床诊断为多囊肾CKD 3-4期的病例，估计需要的样本含量，选择纳入和排除标准的人群，与患者签订知情同意书；患者同意成为我们的研究对象后，会在入组治疗前6个月进行患者肾脏囊肿MRI检查，在抑囊方或中药模拟剂治疗0个月及6个月还需各进行一次肾脏囊肿MRI检查及收集在入组后0个月、3个月、6 个月的的血液和尿液；将样本送检，观察肾脏cyst volume、cyst volume /kidney volume变化和分析血尿指标。还会定期对您随访。您确定参与我们的研究，需要6个月的治疗及观察时间。

**5、参加本研究可能的风险与不良反应**

（1）研究药物可能的副作用、不良反应：本研究会为您提供中药抑囊方或者中药模拟剂服用，可能会造成或加重腹泻的症状。如果您有任何不适，或者病情发生新的变化，或任何意外情况，不管是否与药物有关系，均应及时通知研究医生，研究医生将对此作出判断和医疗处理。

（2）抽血的风险：包括短暂的、轻微的疼痛、局部青紫，少数人会有轻度头晕，或极为罕见的针头感染。

（3）其他风险：还可能存在一些目前无法预知的风险、不适，药物相互作用或不良反应。

**6、参加本研究有什么获益吗？**

您可能不会从本研究中直接获益，但是您的参加将对研究治疗多囊肾CKD3-4期有效的中药复方新方法，填补国内外多囊肾治疗上的空白产生重要作用，有益于人类最终攻克多囊肾疾病。将有助于对疾病作出诊断，为您的治疗提供必要的建议，或为疾病的研究提供有益的信息。

**潜在受益：**本研究可能会阻止/减缓疾病的发展，但是我们不能对此做出保证。尽管参加本次研究可能不会给您带来直接的益处，但您的参加可能会给未来遭受同样痛苦的患者带来益处。

**7、如果不参加此项研究，有没有其他备选治疗方案？**

如果您决定不参加这项研究，您将会得到其他的标准化的治疗（①一般处理：注意休息，避免剧烈的体力活动和腹部撞伤，予以低盐优质低蛋白饮食，必要时予以α-酮酸（开同）口服。②积极治疗各种并发症：控制血压：予以科素亚、钙离子拮抗剂等。血尿急性期：止血敏、立止血等；并发感染：抗感染治疗等）。您的研究者将给您提出适合您的治疗方案。您的研究者也会乐于解释用于治疗您的疾病的其他疗法的可能的好处和风险。

**8、参加该项研究的费用**

您参加本研究，可以免费获得中药抑囊方治疗，并且免费进行3次肾脏MRI、3次肾功能（BUN、Scr、UA）、血常规、尿常规、粪常规+OB、肝功能， 2次尿β2-微球蛋白(β2-MG)、尿视黄醇结合蛋白（RBP）、尿微量白蛋白/尿肌酐（MALB/Cr）、心电图等检测，以上化验及检查费用由本研究承担。但是基础治疗的用药和超出以上说明的检查化验项目需要您自费承担。

**9、发生研究相关伤害的处理**

如果您在研究期间发生了与本研究相关的不良事件并对您造成伤害的，研究者将积极为您治疗，依法承担有关责任。若您未依照研究医生根据本临床试验方案给您的要求，申办方(研究者)将不会承担您的医疗费用。

**10、自愿参加、退出试验**

您可以选择不参加本研究，或者在任何时候通知研究者后退出研究而不会遭到歧视或报复，您的任何医疗待遇与权益不会受到影响。

如果您不能立即做出决定，您有充分的时间考虑，如有需要您可以与亲属、朋友等您信赖的人商量后，再做出决定。

如果您需要其他诊断/治疗，或者您没有遵守研究计划，或者有任何其他合理原因，研究者可以终止您继续参加本研究。

如果决定提前退出研究，您需要咨询研究者还需要遵循哪些程序，这十分重要。试验期间，您可随时了解与本试验中与您有关的信息资料。

**11、您的个人信息会得到严格保护**

如果您决定参加本项研究，您参加研究及在研究中的个人资料均属保密。负责研究医师及其他研究人员将使用您的医疗信息进行研究。这些信息可能包括您的姓名、地址、电话号码、病史及在您研究来访时得到的信息。为确保研究按照规定进行，必要时，研究申办者，药政管理部门或伦理审查委员会的成员按规定可以在研究单位查阅您的个人资料。这项研究结果发表时，将不会披露您个人的任何资料。

**12、其他**

1、出现下列情况时，为了您的健康，研究者可能会未经您的同意而将您撤出本试验：

继续参加本试验，可能会导致您的风险大于受益；

您未按照研究者指导，依照研究方案参加试验；

试验提前终止。

2、我们仍建议您在试验期间采取必要的避孕措施，在试验过程中如果您或您的配偶怀孕，请立即告诉研究者或您的医生。

**13、如果有问题或困难，该与谁联系？**

您可以随时了解与本研究有关的信息资料和研究进展，如果您有与本研究有关的问题，或您在研究过程中发生了任何不适与损伤，请可以联系您的研究医生：______。

电话：	。

如果在研究过程中您有相关权益方面的问题，可以联系我院生物医学伦理委员会，联系电话：	。

**知情同意书·签字页**

**受试者知情同意声明**

我已经阅读了这份知情同意书，并充分理解所有内容。

我有机会提问而且所有问题均已得到解答。

我理解参加本项研究是自愿的。

我可以选择不参加本项研究，我的任何医疗待遇与权益不会因此而受 到影响。

如果我需要其它治疗，或者我没有遵守研究计划，或者发生了与研究相关的损伤或者有任何其它原因，研究医师可以终止我继续参与本项研究。

我将收到一份签过字的“知情同意书”副本。

受试者姓名：__________	法定代理人签名：__________

受试者签名：__________	与受试者关系：	__________

受试者电话：__________	法定代理人电话： __________

日期：	年	月	日	日期：	年	月	日

（注：如果受试者无行为能力时则需法定代理人签名）

**研究者告知声明**

我已经向告知该受试者或其法定代理人详细介绍了该研究的目的、方法、程序及研究的风险和获益等；给与他/她足够的时间阅读知情同意书、与他人讨论，并回答了他/她提出的所有问题；我已经告知该受试者当遇到问题时的联系方式；我已告知该受试者或其法定代理人他/她在研究期间的任何时候无需任何理由退出本研究。

研究者姓名： _______________

研究者签名： _______________

研究者电话： _______________

日期：	年 月 日
