# Supplementary material for: Yinang formulation versus placebo granules as a treatment for chronic kidney disease stages III–IV in patients with autosomal dominant polycystic kidney disease: study protocol for a double-blind placebo-controlled randomized clinical trial
Source: Trials. 2019 Aug 7;20:481. doi: 10.1186/s13063-019-3563-5 (PMC6686499; doi:10.1186/s13063-019-3563-5)
Supplement: Supplementary file 2 — Model consent form given to participants and authorized surrogates. (ZIP 39 kb) [file 13063_2019_3563_MOESM2_ESM.zip › Additional file 2-Consent form in EnglishR2.doc]

Consent form

（Edition 03, September 18, 2018）

Name of subject ： ________________

Binding address ： ________________

Telephone number： ________________

Research center: _______________

**Consent form· Notification page**

Dear Mr. / Madam**___________:**

We sincerely invite you to participate in the " Clinical Trial of Yinang formula for Stages III-IV Chronic kidney disease in patients with Autosomal dominant polycystic kidney disease". This study is conducted under the auspices of the department of nephrology, Shuguang Hospital affiliated to Shanghai university of TCM. Shanghai Changzheng Hospital affiliated to second military medical university and Shanghai Municipal Hospital affiliated to Shanghai university of TCM will be the sub-centers of this study. Before you agree to participate in this study, please read this consent form carefully. The consent form will provide you with the research background, purpose, method, benefits, the possible risk or unchange bring to you during the trial, your rights and interests protection. The information provided in this consent form can help you decide whether to participate in this study or not. If you have any questions, please consult the researchers of the project to ensure that you fully understand the content. If you agree to participate in this study, please sign the consent form and keep a consent form signed by the two sides. This research program has been approved by the ethics committee of all the three centers.

**1. Why participate in this study?**

Autosomal dominant polycystic kidney disease (ADPKD) referred to as polycystic kidney disease (PKD) is one of the most common human hereditary kidney disease, the incidence of PKD is about 1/1000 ~ 1/400 of the world, which has become into the fourth causes of end-stage renal disease (ESRD), there is currently no causative drugs to control the disease progression and the majority of patients survive with hemodialysis, which brings heavy medical and economic burden to patients and society. Adult polycystic kidney disease as autosomal dominant genetic disease, caused by genetic mutations in PKD1 and PKD2. Compared with other races, due to the difference of genome polymorphism, Han gene sequence has its own characteristics in our country, the PKD gene mutations also have its own regular, but the related research is still in the early stage. Generally, the age of onset of ADPKD is 30-40 years old, and more than 50% of patients aged 60 years old enter into ESRD, accounting for about 10% of the etiology of ESRD. There are about 2.7% of hemodialysis patients and 3.8% of peritoneal dialysis ADPKD patients. According to the data statistics of hemodialysis patients in Europe, America and Shanghai region, PKD is the fourth cause of ESRD. There is nearly 17, 000 PKD patients in the United States enter into ESRD each year, costing $600 million.

As a genetic disease caused by gene mutation, the ideal method to treat ADPKD is to replace the mutated gene with normal gene to correct abnormal protein function, but it is not yet clinically possible. In addition, targeted therapy agents for polycystic kidney, such as drugs to inhibit cell proliferation and secretion of cystic fluid are still in the development stage. Renin angiotensin inhibitors (renin angiotensin inhibitors) have become effective drugs for the treatment of polycystic kidney in the 21st century. Renal interstitial fibrosis is also an important risk factor for the development of polycystic kidney. Except conservative treatment, renal drainage combined with decapitation and decompression is also a common method for the treatment of polycystic kidney. A report of 169 cases of PKD patients with decapitation and decompression showed that selective decapitation and decompression for the first and second stage of patients had a good effect on improving symptoms, lowering blood pressure and delaying renal failure. There was no difference in stage 3 patients before and after surgery. Lacking of specific treatment available for ADPKD still confuse physicians and researchers working in nephrology.

We use Yinang formula (YNF) to tonify kidney and spleen, activate blood circulation and remove blood stasis in a holistic manner. Our recent clinical observation of 35 patients with polycystic kidney confirmed that the improvement rate of TCM syndromes was 97.1% after general treatment combined with YNF. The total score was significantly improved after treatment for 3 months, and 6 months, compare with 0 week, and comparison each other in the group were significantly different (*P*＜0.01); after 6 months，the urine NAG, urine RBP, urine albumin/urine creatinine decreased, and the degree of improvement was significantly different (*P*＜0.05). The YNF not only improve the clinical symptoms of PKD patients, but also improve the polycystic kidney glomerular and tubular function related indicators．

The purpose of this study is to explore new drug and new method to delay the progress of PKD, and to evaluate the efficacy and safety of YNF in treating CKD stages III-IV ADPKD patients.

This work is supported by Shenkang 3-year project-Specialized clinical disease five new transformation project (Grant ID: 16CR3021A). This study has been approved by the Ethics committee of each participating hospital. The International Review Board (IRB) of Shuguang Hospital affiliated to Shanghai university of TCM, the research Ethics committee of Shanghai Changzheng Hospital affiliated to second military medical University, the Ethics committee of Shanghai Municipal Hospital affiliated to Shanghai university of TCM. This study complies with the relevant Chinese laws and regulations, the Helsinki declaration and other ethical principles to protect the rights and interests of subjects.

**2. Who can take part in the study?**

（1）Inclusion criteria

①Adult subjects with a diagnosis of ADPKD as stated above and with TCM diagnosis of spleen, kidney deficiency and blood stasis syndrome.

②Patients must be aged 18-75 years.

③15 mL/min/1.73 m2 ≤ eGFR < 60 mL/min/1.73 m2.

④Without taking renal replacement therapy.

⑤Without chronic hepatitis, hepatic dysfunction or cardiovascular diseases or any other life-threatening conditions.

⑥Negative urine or serum pregnancy test within 24 hours prior to administration of YNF and agreement to use contraception throughout the study and for six months after.

（2）Exclusion criteria

①ADPKD is accompanied by proteinuria (>1 g/d) and diabetic patients.

②Learning disability.

③Patients with chronic hepatitis, drug-induced hepatic dysfunction and other hepatic dysfunctions.

④Recent participation in another clinical trial.

⑤Probable, highly likely or definite allergies to the ingredients of the test drug.

⑥Patients with contraindications to magnetic resonance imaging (MRI).

⑦Patients who do not consent to participate in the study.

⑧Patients taking medications likely to affect ADPKD outcomes.

**3. How many people participated in the study?**

The study is expected to recruit 72 eligible patients, participants will be randomly assigned to the YNF group and placebo group in a ratio of 1:1 in each site. Both the YNF and placebo granules (with a dosage of 10% YNF inside) will be indistinguishable from YNF in shape, size, color and packaging. You may be randomly assigned to the YNF group or placebo group (the probability of being assigned to each group is 50%), but you can only enter only one group for treatment and evaluation. No matter which group you will be assigned, you will be given the appropriate basis and symptomatic treatment for your condition.

**4. What is the research procedure？**

We will first screen the cases diagnosed with CKD stages III-IV ADPKD, estimate the required sample size, select the population with inclusion and exclusion criteria, and sign consent form with the patients. After the patient agrees to be the subject of our study, MRI examination of the patient's renal cyst will be conducted 6 months before the enrollment and treatment. MRI examination of the renal cyst will be conducted at 0 month and 6 months after the treatment by YNF or placebo, and blood and urine collected at 0 month, 3 months and 6 months after the enrollment. Observation of renal cyst volume, cyst volume/kidney volume changes and analysis index of blood in the urine after biological sample inspection. You will be followed up regularly. You are confirmed to participate in our study, which requires 6 months of treatment and observation.

**5. Possible risks and adverse reactions associated with participation in this study.**

(1) The possible side effects and adverse reactions of study drugs: this study will provide you with YNF or placebo, which may cause or aggravate the symptoms of diarrhea. If you have any discomfort, new changes in your condition or any unexpected circumstances whether or not related to the drug, you should inform the research doctor in time, and the research doctor will make judgment and medical treatment.

(2) Blood drawing risks: including transient, mild pain, local bruises, mild dizziness in a few people, or extremely rare needle infections.

(3) Other risks: there may also be some other risks, discomfort, drug interactions or adverse reactions that are currently unknown.

**6. What are the benefits of participating in this study?**

You may not directly benefit from this study, but your participation will play an important role in the research of a new effective TCM for the treatment of CKD stages III-IV ADPKD, fill the gap in the treatment of PKD, and contribute to the eventual conquering of PKD. This will help to diagnose the disease, provide necessary advice for your treatment, or provide useful information for the research of the disease.

**Potential benefits:** This study may prevent/slow the progression of the disease, but we cannot guarantee this. Although participating in this study may not bring you immediate benefits, your participation may bring benefits to future patients who suffer from the same pain.

**7. If you don't participate in this study, is there any alternative treatment?**

If you decide not to participate in this study, you will receive other standardized treatment (①General treatment: pay attention to rest, avoid strenuous physical strength activity and abdominal bruises, have low-salt, high-quality and low-protein diet, if necessary, give oral administration of alpha-ketone acid. ②Positively treat the various complications: Blood pressure control: to give coxoya, calcium antagonist, etc. Acute stage of hematuria: hemostatic sensitivity, immediate hemostasis, etc. Concurrent infection: anti-infection therapy, etc.). Your researcher will suggest a treatment plan that suits you. Your researcher will also be happy to explain the possible benefits and risks of other treatments for your disease.

**8. The cost of participating in the study**

You can participate in this study, free access to YNF or placebo, free for three times in MRI, kidney function test (BUN, Scr and UA), blood routine, urine routine and stool routine+ occult blood test, liver function, free for twice in urine β2-microglobulin (β2-MG), microalbumin/creatinine (mALB/Cr), retinol-binding protein (RBP) and electrocardiogram (ECG), etc. The above tests and inspection fees shall be borne by this study. However, the medication for the basic treatment and the examination or test items beyond the above instructions shall be at your own expense.

**9. Management of the occurrence of research-related injuries**

If you suffer any adverse events related to this study or cause you any injury during the study period, the researcher will positively treat you and assume relevant responsibilities according to law. The sponsor (investigator) will not be responsible for your medical expenses if you do not comply with the requirements set forth by the investigator under this clinical trial protocol.

**10. Voluntary participation and withdrawal from the test**

You may choose not to participate in the study, or withdraw consent form from the study after being notified by the researchers at any time without discrimination or retaliation. Your medical treatment and rights will not be affected.

If you cannot make decision immediately, you have enough time to consider, if necessary, you can consult with relatives, friends and other people you trust before making decision.

The investigator may terminate your participation in the study if you require additional diagnosis/treatment, or if you do not follow the study plan, or for any other reasonable reason.

If you decide to withdraw early from a study, it is important that you consult the researchers about what other procedures to follow. During the study period, you may keep in touch with the information related to you in the study.

**11. Your personal information will be strictly protected**

If you decide to participate in the study, your personal information in and about the study will be kept confidential. Responsible for research physicians and other researchers will use your medical information for research. This information may include your name, address, telephone number, medical history, and information obtained during your study visit. To ensure that the research is conducted in accordance with the regulations, the sponsor, the pharmaceutical administration or the members of the ethics review committee are required to have access to your personal data at the research institute when necessary. Your personal information will not be disclosed when the results of this study are published.

**12. Other items**

1. For the sake of your health, the researcher may withdraw you from this study without your consent if:

If you continue to participate in this study, the risks may outweigh the benefits;

You do not participate in the study according to the study protocol instructed by the researcher;

The test is terminated prematurely.

2. We still recommend that you take necessary contraceptive measures during the trial if you or your partner during the trial become pregnant, tell the researcher or your physician immediately.

**13. Who should you contact if you have any questions or difficulties?**

If you have any questions about this study or if you have any discomfort or injury in the course of this study, please contact your research physician: ______。

Telephone: 。

If you have any questions about your rights and interests during the research, please contact the biomedical ethics committee of our hospital at: 。

**Consent form·Signature page**

**Subject consent form statement**

I have read this consent form and fully understand all the contents.

I have the opportunity to ask questions and all questions have been answered.

I understand that participation in this study is voluntary.

I can choose not to participate in this study, and my medical treatment and benefits will not be affected.

If I need additional treatment, or if I do not follow the study plan, or if there is a research-related injury or for any other reason, the study physician may terminate my continued participation in the study.

I will receive a signed copy of the informed consent.

Name of subject: __________	Signed by legal agent: __________

Signature of the subject: __________ Relationship with subjects: __________

Telephone of subject: __________	Legal representative telephone: __________

Date: Year	Month	Day	Date: Year	Month	Day

(Note: legal representative's signature is required if subject is incapacitated)

**Investigator notification statement**

I have informed the subject or his/her legal representative of the purpose, methods, procedures, risks and benefits of the study in detail; Give him/her enough time to read the informed consent form, discuss with others, and answer all questions he/she raises; I have informed the subject of the contact information when encountering problems; I have informed the subject or his/her legal representative that he/she does not need any reason to withdraw from the study at any time during the study.

Name of the investigator: ______________

The investigator's signature: _______________

Researcher telephone: _______________

Date: Year	Month	Day
